# Isokinetic Strength Profile of the Wrist Muscles: A Study of Healthy Women and Men

**DOI:** 10.3390/jfmk10040377

**Published:** 2025-09-30

**Authors:** Smadar Peleg, Eitan Shemy, Michal Arnon, Zeevi Dvir

**Affiliations:** 1 Faculty of Sports and Movement Sciences, The Levinsky-Wingate Academic College, Wingate Campus, Netanya 4290200, Israel; smadarp13@l-w.ac.il (S.P.); eitanshemy@gmail.com (E.S.); michalar@l-w.ac.il (M.A.); 2 Department of Physical Therapy, The Gray Faculty of Medical and Health Sciences, Tel Aviv University, Tel Aviv 6997120, Israel

**Keywords:** wrist, flexion, extension, ulnar deviation, radial deviation, isokinetic

## Abstract

**Objective:** In the isokinetic literature, relatively limited attention has been paid to muscles of the wrist. Therefore, the objective of this study was to present an isokinetic profile of these muscles comprising the flexors (F); extensors (E); and ulnar (U) and radial (R) deviators. **Method:** The dominant-side F, E, U and R in 40 healthy participants (20 women and 20 men) were tested concentrically (Con) and eccentrically (Ecc) using a single speed of 90°/s. **Results:** Men were significantly stronger than women in both the Con and Ecc tests, as indicated by both the absolute (Nm) and the bodyweight-normalized (Nm/kgbw) representations. However, the bodyweight-normalized women/men strength ratio (78.6 ± 8.0%) was significantly higher than the absolute strength ratio (64.1 ± 6.6%). For both the Con and Ecc tests, and irrespective of the representation (absolute or normalized), the U was the strongest muscle group, followed successively by the F, R and E. This rank order was highly significant statistically. The eccentric/concentric strength ratios, E/C_F_ and E/C_U_, were significantly higher in men than in women, with no remarkable inter-sex differences for E/C_E_ and for E/C_R_. A correlational analysis, which included all pairs of basic isokinetic outcome parameters (e.g., the PM of F_con_), was performed with respect to ‘sex’ using a nonparametric bootstrap procedure, revealing that men had significantly higher overall correlation coefficients compared to women. **Conclusions:** The consistency of the main findings with respect to both the sex of the participants and the various strength ratios supports the use of the current protocol. The observed strength order (U > F > R > E) may assist clinicians in setting preliminary return-to-function targets after wrist rehabilitation.

## 1. Introduction

Compared to the characterization of the isokinetic strength of muscles operating on the shoulder and elbow joints, that of the four major muscle groups of the wrist (the flexors (F); extensors (E); and radial (R) and ulnar (U) deviators) has been very limited [1]. This scarcity was indeed highlighted in a recent systematic review of wrist muscle strength research, which included the pronators and supinators [2]. Among other things, this research void, especially in the clinical domain, reflects some inherent issues. For example, isokinetic dynamometers are large, nonportable and expensive instruments compared to the small isometric instruments that may be employed in hand clinics. Furthermore, the complexity of the measurement, exemplified by the multiplicity of parameters that need to be controlled, renders examination using this instrument less applicable to wrist muscles. Specifically, there is no commonly accepted protocol for isokinetic testing of the wrist or grip muscles.

Indeed, the isokinetic testing protocol of any muscle group consists of a number of critical parameters that must be maintained in order for the test findings to be interpretable. Specifically with respect to the muscles of the wrist, the main factors include the segment position, e.g., forearm in neutral vs. pronated or supinated position; body position, e.g., sitting or in standing; the range of segmental motion; the angular speed of joint motion; the speed ramping (low- vs. high-acceleration/deceleration mode); and absence or use of verbal encouragement [1]. Furthermore, the age and sex of the participants are major confounding factors. In particular, the latter has two ramifications. First, strength findings derived from women cannot be compared with those of men. As strongly indicated with respect to the absolute isokinetic strength of the F and E, women are about 60% as strong as men [3,4,5,6,7]. Second, combining sexes can confound interpretation, leading to non-interpretable results, as exemplified by several previous studies [8,9,10,11], and is therefore discouraged unless analyses are stratified. In addition to these hurdles, isokinetic studies of wrist muscle strength, which are based on a small number of participants [4,5,6,7,8,9,10,11], or the use of force (N) rather than moment (Nm) values, likewise preclude interpretation [12].

Most isokinetic studies of wrist muscles focus on the F and E. In terms of sample size (N > 20) and separation of findings according to sex, six studies stand out [3,5,6,7,13,14]. These studies were largely limited to concentric (Con) testing, although the more recent addition of an eccentric (Ecc) component introduced a new dimension and understanding of wrist muscle action [7,15].

In spite of a variety of protocols, in all studies that focused on F and E, the former muscles were stronger than the latter, in both sexes alike, resulting in a maximal moment ratio: F/E of between 1.7 and 2.4. On the other hand, isokinetic assessment of the U and R muscles has been sparsely explored and based on a small number of participants [16,17]. Given the disparity of findings between the two papers and the omission of reference to the test range of motion (RoM) in both, the derived findings from these studies should be viewed with care. Conspicuously, only two studies included isokinetic measurements of all four muscle groups. Ağırman et al. (2017) used a mixed-sex patient group with carpal tunnel syndrome (11 women, 2 men, age range 29–60) and six healthy control subjects (4 women, 2 men, with a similar age range), to test the Con strength of all wrist muscles at 30°/s [18]. However, due to sex pooling, very low baseline muscle strength and a wide age range, this study cannot serve as a valid source. In another study, Chu et al. (2018) [17] followed up on the increase in wrist muscle Con strength in 9 male subjects, who had undergone targeted training, along with 10 controls. In this case, the U strength at baseline assessment was reported as significantly lower than that of the F and on par with that of the E, while post-training, against a fairly homogenous increase of around 40% in strength in the F, E and R, the gain in U strength was extraordinarily (~100%) higher. This finding was neither referred to nor explained in the text, calling into question the accuracy or the reporting of the results.

In addition, a recent study by our group revealed that by correlating the Con and Ecc strengths of the F and E, men had a significantly (*p* = 0.003) higher overall inter-muscular correlation values compared to women [7]. Whether this inter-muscle strength pattern (IMSP) is maintained upon adding the U and R could not be predicted, based on the uniqueness of this finding. Thus, extending the analysis to all four muscle groups became an additional objective.

Finally, from the clinical viewpoint, it should be noted that profiling the four wrist muscle groups in the same session allows for a fast assessment of the wrist as an integral functional unit. Therefore, the objectives of this study were threefold: (1) derive the maximal isokinetic strength profile (Con and Ecc) of the wrist F, E, U and R in healthy women and men; (2) establish a strength paradigm which depicts the inter-muscle relationships in terms of both the absolute (Nm) and bodyweight normalized (Nm/kgbw) units; (3) explore the IMSP as it applies to all four muscle groups operating on the wrist.

## 2. Materials and Methods

### 2.1. Participants

Forty healthy participants, 20 women and 20 men, who were students and staff at the Levinsky-Wingate Academic College, were recruited via social media and noticeboards. The mean (SD) age (y), stature (cm), weight (kgf) and BMI of the women were 25.8(2.9), 163(6.0), 59.1(8.9) and 22.3(3.0), respectively, while the parallel values for men were 25.5(2.3), 176.3(7.3), 74.3(9.9) and 23.8(2.5). All participants were right-hand dominant; none were involved in activities that put a large load on the hands (wrist and fingers), e.g., rock climbing; none had a medical history relating to the forearm-hand region; and none of the women were pregnant during the time of testing. This study was approved by the IRB of the Levinsky-Wingate Academic College (Wingate Campus), N. 414, 13 July 2023.

### 2.2. Experimental Procedure

#### 2.2.1. Instrumentation

Participants’ stature and weight were measured using a stadiometer (Tanita Corp., Tokyo, Japan) and a calibrated digital scale (Lifebl312, Canny Corp., Beijing, China), respectively. The isokinetic strength measurements were carried out using a Biodex System 4 Pro isokinetic dynamometer (Shirley, NY, USA), which was calibrated prior to the commencement of the study.

#### 2.2.2. Test Protocol

**Figure 1:**
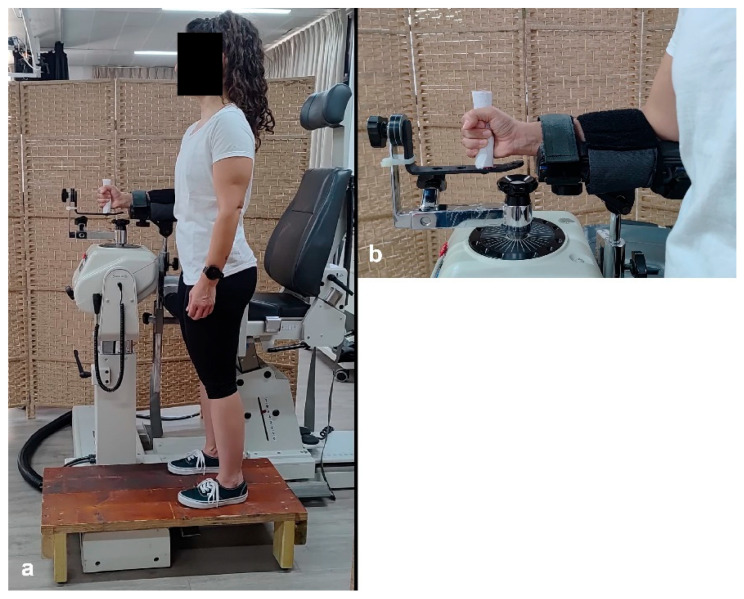
Testing position of wrist flexion and extension (**a**), and a close-up of forearm support and straps (**b**).

**Figure 2:**
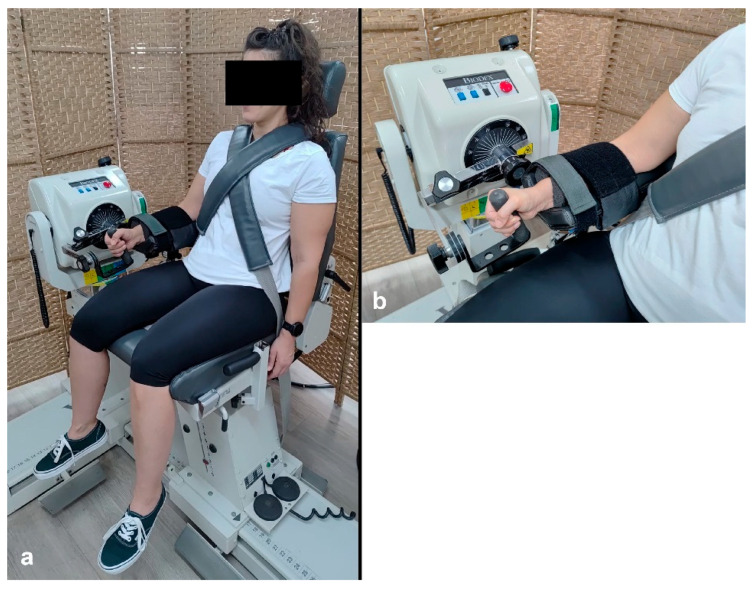
Testing position of wrist ulnar and radial deviation (**a**), and a close-up of forearm support and straps (**b**).

Participants were instructed to avoid any strenuous upper limb activity, including training, during the 48 h preceding the test session. Unilateral (right side) Con and Ecc strengths of the wrist F, E, U and R were measured in a single session. For both F-E and R-U testing, the dynamometer axis was aligned with the radiocarpal joint. No correction for wrist weight was applied, as the gravitational effect was negligible [19]. Due to the mechanical constraints of the dynamometer, different body positions were required for testing in different movement planes. F–E was tested in a standing position, with the dynamometer’s shaft oriented vertically, as this setup required lateral access and could not be performed while seated (Figure 1a). Therefore, wrist motion took place in the horizontal plane, i.e., with the dynamometer shaft in vertical position [7,19]. On the other hand, testing U and R was conducted in a sitting position, with the dynamometer oriented horizontally (Figure 2a). In both setups, the forearm was stabilized in a neutral (mid-pronation/supination) position on a cushioned support using two straps, one close to the elbow and one close to the wrist (Figure 1b and Figure 2b). For U and R testing, a backrest and two crossed shoulder straps were used to minimize trunk and shoulder movement. During the standing position, participants whose height was lower than 180 cm were asked to stand on a custom-built pedestal to ensure a comfortable elbow flexion angle of 60–75°. During the familiarization phase conducted before each testing session, the participants were instructed to remain upright and initiate movement only from the wrist, while receiving feedback to correct any compensatory movements, e.g., tilting their upper body or shoulder abduction.

During all 4 movement patterns, participants gripped a cylindrical handle extending from the dynamometer’s lever arm, positioned at an individually adjusted distance. They were instructed to grip the handle throughout the full range of motion, in both the Con and Ecc efforts. Grip force was not constrained or quantified, but subjects were told to focus on producing maximal force in the wrist muscles. Consistency was ensured by monitoring the coefficient of variation (CV%), with all accepted tests kept below a 10% threshold. Additionally, since proper Ecc engagement in isokinetic mode requires sustained force in the same direction at the end of the Con phase, participants who failed to meet this pattern were asked to repeat the familiarization trial.

In both tests, the RoM spanned 45°; from 5° of E to 40° of F in F-E, and from 5° of R deviation to 40° of ulnar deviation in R-U. Angular speed was set at 90°/s for all tests. The location of this test RoM ensured that during eccentric testing, the initial moment produced by the tested muscle group was sufficient to exceed the threshold level required by the dynamometer’s operational characteristics. As for the test speed, 90°/s was determined following a pilot study, which proved the latter to be well tolerated for all 4 movement patterns. As for the relatively short RoM, a series of studies using isokinetic short range of motion (SRoM) vs. commonly employed RoMs had supported the application of the former in healthy participants as well as in patients in different joints, including the wrist [11,12,13]. In addition, all individual isokinetic curves were inspected during the tests to ensure that the peak moment (PM) in each of the muscle groups was reached within the specified RoM.

Following a general warm-up, participants performed a familiarization session before each of the first two movement pairs (F_con_-F_ecc_ and E_con_-E_ecc_), consisting of 3 increasing exertions (50%, 70% and 90% of maximal intensity). For the second two movement pairs (U_con_-U_ecc_ and R_con_-R_ecc_), familiarization included only two repetitions (50% and 70% maximal intensity) since adding a 90% submaximal intensity repetition induced fatigue as demonstrated by a pilot study. Actual testing was commenced 90 s after the familiarization and was based on 3 reciprocal pairs of maximal Con and Ecc contractions of each of the four muscle groups. A rest of 5–10 s was allowed between each individual repetition, while 2–3 min were allowed between F and E and U and R tests. The time for repositioning between the sitting and standing positions was around 15 min. All participants performed the tests in the same sequence: F_con_-F_ecc_, E_con_-E_ecc_, U_con_-U_ecc_ and R_ecc_-R_con_. No verbal encouragement was given by the examiner during testing.

### 2.3. Statistical Analysis

Data analysis was performed using the SPSS (v. 29.0) program. The maximal individual strength value—PM (in Nm)—out of the 3 maximal repetitions for each individual muscle, movement and contraction mode was used as the representative outcome score. Significance was set at *p* < 0.05. For descriptive statistics, quantitative variables were described using the mean (±SD). The following four ratios were calculated: within-muscle Ecc-to-Con ratio (E/C), namely E/C_F_, E/C_E_, E/C_U_ and E/C_R_ 2; between antagonistic muscles but the same contraction mode, namely F/E_con_, F/E_ecc_, U/R_con_ and U/R_ecc_. Each PM score was examined for normality assumption via skewness (*SK* < [2.0]) and kurtosis (*K* < 7.00) procedures. Skewness values ranged between −0.15 and 1.28, and kurtosis values ranged between (−1.03) and (+3.70), justifying the assumption of normal distribution for all variables [20].

An independent *t*-test was conducted to compare the PM values and the ratio values between men and women. A three-way ANOVA with repeated measures was conducted on PM variables, with two within factors as follows: 4 muscles × 2 contraction modes and one between factor (sex). This analysis revealed two noteworthy results: a significant interaction between sex and the 2 main effects (muscle group, contraction mode), which indicated that women and men reacted differently in each condition. In addition, there was a significant difference between the sexes with a high effect size, η^2^ = 0.62. Therefore, a 2-way ANOVA with repeated measures model of the form: 4 muscles × 2 contraction modes was used for each sex separately. For the calculated ratios, a Mixed ANOVA was applied with one repeated variable (muscle) and sex as a between-subject variable. To compare women vs. men in terms of overall correlation patterns among the PMs, pairwise correlations within each group (men or women) were estimated using a nonparametric bootstrap procedure with 1000 resamples. For each coefficient, we report bias-corrected and accelerated (BCa) 95% confidence intervals. To assess whether the overall structure of associations differed between men and women, we conducted two complementary global tests: Jennrich’s test, which evaluates whether two correlation matrices are statistically equivalent, and the Box’s M test, which compares the equality of covariance matrices between groups and serves as a robustness check under different distributions.

Sphericity and Multiple Comparisons Control: for all repeated-measures ANOVA analyses, Mauchly’s test of sphericity was conducted to assess the assumption of sphericity. When sphericity was violated (*p* < 0.05), the Greenhouse-Geisser correction was applied to adjust degrees of freedom and *p*-values. For post hoc pairwise comparisons within repeated measures analyses, the Bonferroni correction was applied to control for multiple comparisons. For independent *t*-tests comparing groups, Cohen’s d or Hedges-g effect sizes were calculated and reported alongside significance tests.

## 3. Results

None of the participants complained of inconvenience or pain during or following the tests. The mean (±SD) values of the Con and Ecc absolute and bodyweight normalized strength of the F, E, U and R, and the sex-based strength ratio of women vs. men, PM_W_/PM_M_ (expressed in %), are outlined in Table 1.

An independent sample *t*-test was conducted to examine the between-sex differences in terms of absolute and normalized strength. The results indicated that men were significantly stronger than women, by both representations, across all measured muscle groups, with the exception of the normalized Con strength of F and U. To examine the difference between the ratios, PM_W_/PM_M_ (absolute and normalized), a paired samples *t*-test revealed that the normalized ratio (78.6 ± 8.0%) was significantly higher than the absolute’s (64.1 ± 6.6%) with a mean difference of 14.9 ± 1.1% (t[7] = 29.00, *p* < 0.001, Cohen’s *d* = 10.25).

**Figure 3:**
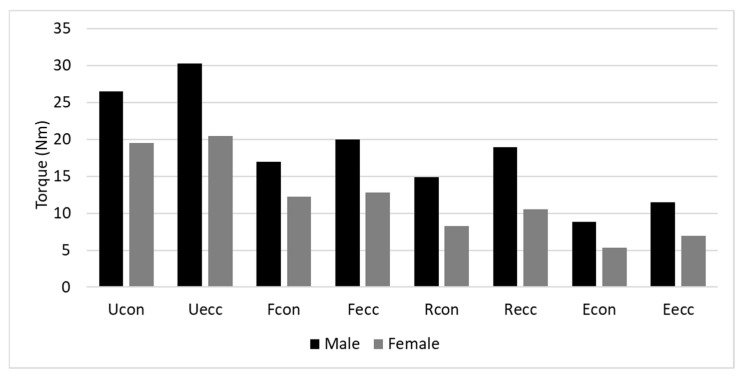
Wrist muscle strength profile. Index: F—flexors, E—extensors, U—ulnar deviators, R—radial deviators.

**Figure 4:**
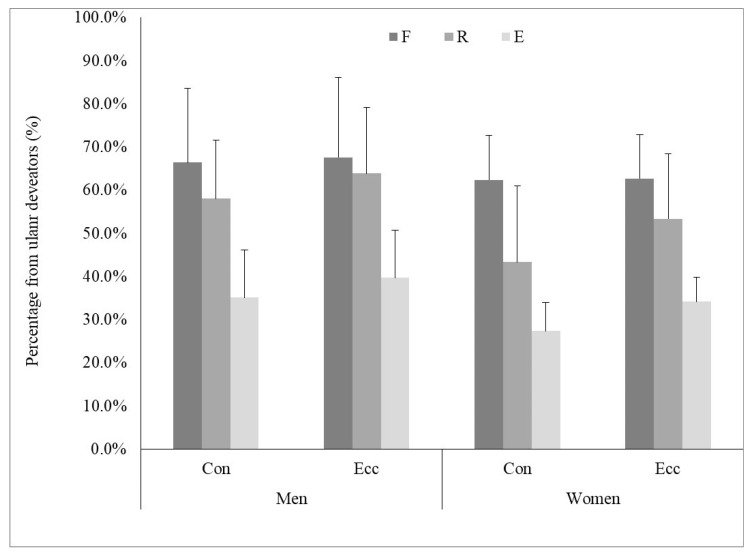
The strength (PM) of the flexors, radial deviators and extensors is expressed as a percentage of the ulnar deviators. Index: Con—concentric, Ecc—eccentric, E—extensors, F—flexors, R—radial deviators.

Regarding the strength order of the four muscle groups, irrespective of contraction mode (Con or Ecc) or representation (absolute or normalized), the U was the strongest, followed successively by F, R and E, as shown by the bar graph (Figure 3). To gain further insight into the inter-muscle relationship, the PM values of the F, E and R were expressed as a percentage of the U (Figure 4). A three-way mixed ANOVA was conducted on the relative values, including two within-subject factors (‘muscle’ and ‘contraction mode’) and one between-subject factor (‘sex’). The analysis revealed statistically significant differences for all these main effects. Regardless of the first two factors, men demonstrated significantly higher values compared to women: 55.1% ± 9.4% vs. 47.2% ± 9.4%, respectively, F(1,38) = 7.10, *p* < 0.01, η^2^ = 0.156. Ecc contractions yielded significantly higher values than their Con counterparts: 53.6% ± 1.7% vs. 48.8% ± 1.4%, respectively, F(1,38) = 63.38, *p* < 0.01, η^2^ = 0.625. For the muscle group main factor, the F(64.8% ± 14.9%) produced significantly higher values than the R (54.6% ± 10.9%), F(1,38) = 12.50, *p* < 0.001, η^2^ = 0.247, which in turn were associated with significantly higher values than the E (34%.1 ± 6.2%), F(1,38) = 75.62, *p* < 0.001, η^2^ = 0.666.

Additionally, a significant interaction between contraction mode and muscle group was observed: F(2,76) = 26.61, *p* < 0.01, η^2^ = 0.412. Post hoc analyses revealed that Ecc contractions yielded significantly higher values compared to Con contractions for R (58.6% ± 11.2% vs. 50.7% ± 10.8%, *p* < 0.01) and E movements (36.9% ± 6.4% vs. 31.2% ± 6.2%, *p* < 0.01), while no significant difference was found for F movement (65.2% ± 10.0% vs. 64.4% ± 10.5%, *p* = 0.319). Finally, sex-related differences varied as a function of muscle group and contraction mode. Men demonstrated significantly higher relative strength than women in both contraction modes for R and in Con contractions for E, while no significant sex differences were observed for F muscles across either contraction mode (Figure 4).

Using a two-way ANOVA with repeated measures, two types of ratios were examined: (1) within ‘muscle’—between ‘contraction’, namely E/C_F_, E/C_E_, E/C_U_ and E/C_R_; (2) between ‘muscle’—same ‘contraction’, namely: F/E_con_, F/E_ecc_, U/R_con_ and U/R_ecc_. The values for the above ratios are outlined in Table 2. As for the first series of ratios, the main findings revealed that the E/C_F_ and E/C_U_ were significantly higher (*p* < 0.001, *p* < 0.001) in men than in women, with no remarkable inter-sex differences for E/C_E_ and for E/C_R_. Moreover, for both sexes, the E/C_U_ was the lowest ratio. With respect to the second series of ratios, the pairwise comparison revealed that the ratio values for men were significantly lower (*p* < 0.001, *p* < 0.01) in U/R_con_ and in U/R_ecc_, compared to those of women, with no remarkable inter-sex differences for F/E_con_ and F/E_ecc_. The analysis also revealed that in both women and men, F/E_con_ was significantly higher (*p* < 0.001, *p* < 0.001) than F/E_ecc_, while U/R_con_ was significantly higher than U_ecc_/R_ecc_ (*p* < 0.05, *p* < 0.001). Furthermore, the results show that the order of the ratio values for men and women was generally similar, with some minor differences.

Finally, the bootstrapped correlations with BCa 95% confidence intervals (presented in Table 3 and in Supplementary Material Table S1) indicated consistently stronger and more homogeneous associations among men compared with women. Global comparison confirmed these descriptive differences. The Jennrich’s test indicated a significant overall difference between men’s and women’s correlation matrices, χ^2^(28) = 77.3, *p* < 0.001. Likewise, Box’s M test supported this conclusion (M = 163.2, *p* < 0.001), suggesting that the covariance structures also differ reliably between sexes. Taken together, these findings suggest that the inter-muscle strength pattern (IMSP) indices were considerably stronger and more uniform in men compared with women.

## 4. Discussion

This study focused on the Con and Ecc strength of the wrist muscles and their sex-related variations. The main finding is that, for both contraction types and irrespective of sex, the strongest wrist muscle group is the U, followed successively by the F, R and E. In addition, based on the inter-muscular correlations, women and men differ significantly in what may be termed as the inter-muscle strength pattern (IMSP).

The significance of the wrist muscles’ strength order lies in both the anatomical–biomechanical and the clinical domains. While the former is primarily a reflection of the force vectors of the operating muscles and their associated lever arms, the latter could provide an additional guideline in the rehabilitation of wrist injuries, especially in the presence of muscle strength deficiency. Given the particular importance of both, a preliminary assessment of the validity of the strength order is necessary. Such an assessment may be approached through three different venues. One, which is based on comparison to previous findings relating to all four muscle groups, was ruled out since the available studies could not serve this purpose for the reasons highlighted above [17,18]. Therefore, a more limited approach is to compare to F and E strengths, which were explored in studies that related to both sexes, employed a substantially similar test protocol, and applied the same dynamometer. Close similarity between the former and the current findings, albeit relating to only 2/4 patterns, would provide a preliminary basis for the validity of the strength order. However, prior to such a comparison, an important qualifying factor should be considered. With one exception, all past studies relating to wrist muscle strength placed the hand of the tested side in a grip configuration [15]. This is, in fact, a direct result of the design of all wrist muscle-testing attachments serving in any of the commercially available isokinetic dynamometers, namely the use of a forearm support that provides proximal stabilization and a handle that allows a solid grip during the test. Thus, the recorded isokinetic strength reflects, at least with respect to wrist F, the cumulative effect of the actual wrist flexors as well as that of the muscles responsible for the grip.

A number of studies that quoted sex-based values and used the same angular speed (90°/s) can serve this purpose. A mean (±SD) Con PM of the F of 14.6 ± 3.4 Nm (0.26 ± 0.06 normalized) and 26.7 ± 4.5 (0.35 ± 0.04) in women and men, respectively, has been reported [3]. The respective figures for E were 6.9 ± 1.8 (0.12 ± 0.03) and 10.5 ± 1.8 Nm (0.14 ± 0.02). In another study relating to the age decade 20–29, the PM for F in women was 10.7 ± 2.4 and 18.4 ± 3.8 in men vs. E strength of 5.7(1.4) and 9.4 ± 1.6 Nm, respectively [3]. In a similar study of isokinetic strength in both sexes, which was spread over a wide age range, the F strength in women (N = 72) was 13.1 ± 2.8, whereas for men (N = 90) it was 22.1 ± 5.5 Nm. The respective values for E were 6.0 ± 1.7 and 11.4 ± 3.1 Nm [6]. Finally, in a recent study which employed a slightly higher speed (120°/s) and two groups of women and men (totally different from the current study), the F strengths were 10.7 ± 2.0 Nm (0.18 ± 0.04 Nm/kgbw) and 17.9 ± 3.6 (0.24 ± 0.05) in women and men, respectively. The parallel values for E strengths were 5.84 ± 1.6 (0.10 ± 0.02) and 9.4 ± 2.5 (0.13 ± 0.03 Nm/kgbw) [7]. Thus, except for one study whose mean values exceeded the current findings systematically by about 25%, the other reported values closely align with those derived from the present study. As for the F/E_con_ ratio, the present values of 2.05 ± 0.76 and 2.34 ± 0.48 in men and women, respectively, should be compared with those obtained by Forthomme et al. (2002) [3] (2.17 ± 0.63 (men) and 2.55 ± 0.29 (women)) and Harbo et al. (2012) [6] (1.94 and 2.2, respectively), indicating good to excellent agreement among the studies.

The second venue relates to the internal E/C ratios. When applying the same speed in maximal effort testing, the E/C ratio is >1, a physiological variant that has been reported in practically all isokinetic studies to date [21]. In this study, all ECRs were >1, ranging from 1.04 to 1.32. Of note, the higher scores were calculated for E/C_E_ and E/C_R_ with almost perfect inter-sex similarity. For F and U, the values were lower in both sexes and significantly lower still in women compared to men. One possible explanation for this variation is the relatively higher Con vs. Ecc strength associated with the F and U muscle groups, which could possibly result from a higher exposure to concentric powerful contractions of these muscles.

The third venue relates to the inter-sex strength ratios—PM_W_/PM_M_. In particular, the absolute (Nm) ratios ranged from 0.57 to 0.75, with a mean value of 0.66 and 0.62 for the Con and Ecc PMs, respectively, and an overall mean of 0.64. In a recent comprehensive review that principally assessed the sex-related aspects of muscle strength and endurance, the global (all muscles) Con PM_W_/PM_M_ ratio stood at around 0.6 [22]. Specifically with respect to the wrist F and E, and based on the studies by Harbo et al. and others (2012), this ratio was on average 0.6 for the F and slightly smaller for the E, around 0.55 [6]. No parallel figures were quoted for either the R or the U [6]. In another study, the calculated values of the PM_W_/PM_M_ ratio for Con isokinetic efforts at 30 and 90°/s and Ecc at 60°/s were 0.6, 0.55 and 0.58, respectively [3]. The slight differences between the present findings and those derived from the abovementioned studies may also be the result of the difference between the test position of the hand, in the gravitational vs. the nongravitational planes, in the former and latter, respectively. However, probably the most valid comparison is to the findings derived from a previous study carried out by our group on a different group of same age range participants in which the only difference was in the testing speed: 120 instead of 90°/s. The absolute PM_W_/PM_M_ ratio was 0.6 for F_con_, F_ecc_ and E_ecc_ and 0.62 for E_con_, and thus in excellent agreement with the current findings. The increase in the normalized (Nm/kgbw) PM_W_/PM_M_ ratio by around 15% has been highlighted in a recent systematic review with particular reference to wrist muscles [2]. This aligns with parallel findings relating to other muscles in the body [23], while underlining the importance of bodyweight and sex in the predictive equation of strength (see, e.g., [24]). In light of this finding, we recommend the parallel use of absolute and normalized isokinetic strength scores.

Hence, the collective evidence is consistent across analyses and in line with selected prior reports (especially for F/E). Moreover, in spite of the serious dearth of data relating to U and R, we suggest that the strict adherence to the protocol in all possible aspects, the relatively long inter-position break (15 min), which allowed sufficient recovery from the preceding short isokinetic efforts, and the fact that testing was completed in a single session together support the observed isokinetic strength order of the wrist muscles. Given the critical importance of the test protocol, we recommend that, for the sake of standardization, future isokinetic studies of these muscles be conducted under the same test conditions.

In trying to elucidate the underlying factor/s for the strength order, it is worth considering that ultimately, two elements take part in generating a moment around an axis: the magnitude of the force and its moment arm (MA). In biological terms, these are embodied, respectively, by the tension (in N) developed in the relevant muscle and its direction (in angular values) relative to a fixed coordinate system, and the distance (in mm) between that vector and the axis around which the 3D joint motion takes place. It is noteworthy to mention that the magnitude of the vector depends on the invariable muscle/s mass, physiological cross-sectional area (PCSA) and fiber length [25], as well as on the level of activation, relative to the maximum, which most commonly is defined in terms of maximal isometric contraction. Thus, studies on human cadavers, which typically relate to the muscle/s’s PCSA but do not include MA data, are of limited importance in the present context [26,27,28]. Of note, the data presented in these studies, which were based on diverse yet very small samples, indicated significant variations, although the PCSA and masses of U and F muscles were quite similar, with differences of 1–11%. Quite surprisingly, the PCSA and mass of the E were the highest, but without reference to the associated MAs, the strength order of the muscles could not be estimated. This was achieved in a study that tested the isometric strength of the four muscle groups using a custom-made dynamometer instrumented with a rotating load cell whose axis was aligned with the capitate bone [29]. The mean peak isometric flexion moment was 12.2 ± 3.7 Nm, while the parallel values for E, U and R were 7.1 ± 2.1, 9.5 ± 2.2 and 11.0 ± 2.0 Nm, respectively. Thus, not only were the absolute isometric strength values much lower than those obtained in the present study, but the strength order of the muscles was different: F > R > U > E. Significantly, by estimation, F strength was greater than that of U by around 30%, whereas in the current study, U > F by more than 50%.

One possible explanation for the disparity in the findings relates to the differences in the MAs caused by a substantially different location of the center of rotation (CoR). Specifically aligning the CoR with the capitate for the isometric measurements [29] vs. the use of the radiocarpal joint’s CoR in the present study may seriously affect the findings. Based on radiological data extracted from Indian women and men, the distance between the middle of the capitate and the radiocarpal joint is around 20 mm [30]. Furthermore, the CoR during isokinetic motion is constantly varying, whereas in maximal isometric exertions, the CoR would be stationary once the bones are in a tight position. In addition, the test position was different: neutral (midway between pronation–supination) vs. supinatory in the present and the Delp et al. studies, respectively, which probably affected the recorded findings [25]. Indeed, as demonstrated before in a study focusing on the estimation of the wrist joint’s CoR, the method applied for this purpose may have a massive effect on the results [31]. As well, the models and the experimental setups have limited the analysis to the six ‘wrist only’ muscles while leaving the extrinsic muscles operating on the fingers outside the picture in spite of their role in the actions explored [15]. However, to actually test the effect the varying MAs may have on the present results would require online imaging or noninvasive marking of the bones comprising the wrist while monitoring the tendon-associated vectors and level of activity in the muscles that operate on the wrist of live subjects during the isokinetic test. Such a task would be profoundly challenging, at the slightest, from the technical point of view. Alternatively, modeling of this system could serve this objective and shed some light on the major factors taking place in producing the moments recorded in this study. Equally, further research that is based on different isokinetic dynamometers, the same alignment axes and more diverse protocols, e.g., including isometric measurements and additional speeds, could assist in obtaining a comprehensive strength profile of the wrist muscles.

The other major finding emerging from this study indicated that men had significantly (*p* = 0.003) higher overall inter-muscular correlation values compared to women. The same inter-muscle strength pattern (IMSP) was observed for the first time in a recent study conducted by our group [7], which was limited to exploring the Con/Ecc strength of the F and E in both sexes. In the present study, this finding was expanded to include the U and R. A comprehensive literature search failed to produce a similar finding that would highlight the inter-sex differences. However, as the IMSP may reflect a higher demand for coherent muscular action, a possible explanation may be provided by a recent review, which indicated that participation in strength training was significantly higher in men than in women, with a particular emphasis on upper-body exercise [24]. The latter could reflect on the strength of the wrist muscles, but it should be noted that the IMSP is not about the strength per se, absolute or normalized, but on the strength relationships among the muscles.

In the absence of relevant previous research, we speculate that the more pronounced IMSP in men compared to women is probably a result of the need imposed on the muscles to increase the level of muscular co-activation in order to produce higher moments about the wrist. Significantly, none of the four movement patterns is a product of a single muscle; rather, movement against resistance requires co-contraction of two or three out of the six muscles operating on the wrist, according to the following direction: for U—flexor carpi ulnaris (FCU) + extensor carpi ulnaris (ECU); for F—FCU + flexor carpi radialis (FCR); for R—FCR + extensor carpi radialis longus (ECRL) + extensor carpi radialis brevis (ECRB); and for E—ECU + ECRL + ECRB. In order to maximize the moment output, these muscles have to work together, and the higher the demand, the higher the level of co-contraction, as is well established [32,33]. Of note, any pair of muscles in the above has one directional movement in common and one that is opposite. For example, to produce forceful U, FCU and ECU are coactivated, but while they are agonists for U, the first is also a flexor (FCU), and the second (ECU) is an extensor. The other three movement actions demonstrate the same principle. This simultaneous agonist–antagonist action pattern, which occurs more often and at a higher intensity in men compared to women, most probably leads to the significantly higher IMSP in men. Clearly, the correct way to test this hypothesis is by using EMG or co-activation metrics, and therefore future research on the IMSP should integrate EMG and isokinetics within a single framework. Moreover, additional studies focusing on the within- and between-sex differences in IMSP relating to lower and upper extremity muscles that operate multiple planes of motion joints are needed to shed further light on this challenging relationship.

The findings of this study are qualified by three limitations. The first is a technical one: changing body position in F/E vs. U/R testing due to the need to keep the forearm in neutral position during the former’s testing is a recognized limitation. It may be solved using a special seat that is placed next to the dynamometer but which does not interfere with its action. The second relates to the actual protocol, which consisted of a single speed, short RoM, dominant side only, no gravity correction and absence of reliability estimates (ICC/CV), which could affect the generalizability of the derived findings. Attention should be given to these elements in future studies of wrist muscle strength. The third relates to the effect grip force has on the outcome measures. However, this requires an additional sensor, especially a display option, attention to which could interfere with the actual production of maximal moment output in the wrist muscles. Thus, although extrinsic finger flexors may have contributed, especially to F and U moments, we view this limitation as a relative one and recommend applying the same protocol, i.e., focusing on the production of maximal strength in the wrist muscles, without special instructions regarding the force level.

## 5. Conclusions

In this cohort and protocol, U was strongest, followed by F, R, and E, across concentric/eccentric modes. This relative ordering, together with sex-specific ratios, may offer a preliminary guide for clinical goal-setting, pending replication across speeds, ROMs and positions.

## Figures and Tables

**Table 1:** Absolute (Nm) and normalized (Nm/kgbw) isokinetic concentric and eccentric strength values of the wrist flexors, extensors, ulnar and radial deviators in women and men.

Variable	Men				Women				PM_W_/PM_M_							
	Abs	Nor	95% CI		Abs	Nor	95% CI		Abs	Hedges_g	95% CI		Nor	Hedges_g	95% CI	
F_con_	17.0 ± 4.1	0.23 ± 0.06	15.49	18.52	12.1 ± 2.4 **	0.20 ± 0.04	10.63	13.65	71 *	1.428	0.734	2.107	88 *	0.581	−0.044	1.198
F_ecc_	20.0 ± 4.6	0.27 ± 0.06	18.30	21.62	12.7 ± 2.5 **	0.21 ± 0.04 **	11.04	14.36	64	1.937	1.182	2.675	78	1.145	0.479	1.798
E_con_	8.8 ± 1.7	0.12 ± 0.03	8.13	9.43	5.3 ± 1.1 **	0.09 ± 0.02 **	4.67	5.97	61	2.367	1.551	3.164	75	1.281	0.603	1.946
E_ecc_	11.5 ± 1.9	0.16 ± 0.03	10.77	12.22	6.9 ± 1.2 **	0.12 ± 0.02 **	6.15	7.60	60	2.837	1.949	3.707	74	1.456	0.759	2.138
U_con_	26.5 ± 6.6	0.36 ± 0.09	23.76	29.19	20.0 ± 5.3 **	0.33 ± 0.06	17.26	22.69	75	1.062	0.403	1.708	91	0.377	−0.238	0.988
U_ecc_	30.3 ± 7.1	0.41 ± 0.10	27.50	33.08	20.7 ± 5.1 **	0.34 ± 0.06 *	17.92	23.50	68	1.522	0.818	2.211	83	0.843	0.202	1.474
R_con_	14.9 ± 3.4	0.20 ± 0.05	13.42	16.34	8.5 ± 3.0 **	0.14 ± 0.05 **	7.03	9.94	57	1.945	1.189	2.684	70	1.108	0.445	1.758
R_ecc_	18.9 ± 3.9	0.26 ± 0.06	17.29	20.53	10.7 ± 3.2 **	0.18 ± 0.06 **	9.07	12.32	57	2.245	1.447	3.024	70	1.199	0.528	1.856

* (in integer %), Abs—absolute, Nor—bodyweight normalized, con—concentric, ecc—eccentric, F—flexors, E—extensors, U—ulnar deviators, R—radial deviators, PM—peak moment, W—women, M—men, * *p* < 0.01, ** *p* < 0.001 for between sex comparison.

**Table 2:** PM-based isokinetic ratios for wrist muscles in women and men.

Variable	Men	Women	t	*p*	Cohen’s d
E/C_F_	1.18 ± 0.1	1.04 ± 0.05	5.24 **	0.00	1.66
E/C_E_	1.32 ± 0.1	1.31 ± 0.14	0.25	0.80	0.08
E/C_U_	1.15 ± 0.08	1.04 ± 0.05	5.23 **	0.00	1.66
E/C_R_	1.28 ± 0.09	1.32 ± 0.31	−0.58	0.57	−0.18
F/E_con_	2.05 ± 0.76	2.34 ± 0.48	−1.45	0.15	−0.46
U/R_con_	1.84 ± 0.47	2.58 ± 0.88	−3.36 **	0.00	−1.06
F/E_ecc_	1.80 ± 0.55	1.87 ± 0.3	−0.48	0.63	−0.15
U/R_ecc_	1.64 ± 0.37	2.05 ± 0.60	−2.61 **	0.01	−0.82

E/C_F, E, U, R_—eccentric to concentric ratio of the F, E, U and R, respectively; F/E_con_—concentric F to E ratio, F/E_ecc_—eccentric F to E ratio, U/R_con_—concentric U to R ratio, U/R_ecc_—eccentric U to R ratio; ** *p* < 0.001, for between sex comparison.

**Table 3:** Peak moment correlation (Pearson’s r) matrix: women vs. men. Each cell in the matrix includes the r value in women (top) and men (bottom).

Women								
Men	Fcon	Fecc	Econ	Eecc	Ucon	Uecc	Rcon	Recc
Fcon	1.0							
Fecc	0.929 **	1						
	0.978 **							
Econ	−0.3	−0.15	1					
	0.554 *	0.538 *						
Eecc	−0.3	−0.23	0.947 **	1				
	0.693 **	0.677 **	0.886 **					
Ucon	0.500 *	0.467 *	−0.14	−0.05	1			
	0.702 **	0.666 **	0.590 **	0.625 **				
Uecc	0.488 *	0.515 *	−0.11	−0.03	0.966 **	1		
	0.714 **	0.691 **	0.551 *	0.598 **	0.990 **			
Rcon	0.3	0.25	−0.07	−0.1	0.35	0.38	1	
	0.3	0.26	0.30	0.35	0.41	0.39		
Recc	0.3	0.21	−0.05	−0.08	0.38	0.4	0.96 ***	1

* *p* < 0.05, ** *p* < 0.01, *** *p* < 0.001. Index: con—concentric, ecc—eccentric, F—flexors, E—extensors, U—ulnar deviators, R—radial deviators.

## Data Availability

The datasets associated with the present study are available from the corresponding author upon reasonable request.

## Supplementary Materials

The following supporting information can be downloaded at: https://www.mdpi.com/article/10.3390/jfmk10040377/s1, Table S1: 95% Confidence interval (CI) for PM correlation (nonparametric bootstrap procedure) matrix: women vs. men. Each cell in the matrix includes the r value and the 95% CI in women (top) and men (bottom).

## Abbreviations

The following abbreviations are used in this manuscript:

Con	concentric
CoR	Center of Rotation
E/C_E_	PM-based eccentric to concentric ratio of the extensors
E/C_F_	PM-based eccentric to concentric ratio of the flexors
E/C_R_	PM-based eccentric to concentric ratio of the radial deviators
E/C_U_	PM-based eccentric to concentric ratio of the ulnar deviators
E	extensors
Ecc	eccentric
F	flexors
F/E_con_	concentric PM-based flexors to extensors ratio
F/E_ecc_	eccentric PM-based flexors to extensors ratio
MA	moment arm
PCSA	physiological cross-sectional area
R	radial deviators
RoM	Range of Motion
U	ulnar deviators
U/R_con_	concentric PM-based ulnar to radial deviators ratio
U/R_ecc_	eccentric PM-based ulnar to radial deviators ratio
