# Supplementary material for: Isokinetic Strength Profile of the Wrist Muscles: A Study of Healthy Women and Men
Source: J Funct Morphol Kinesiol. 2025 Sep 30;10(4):377. doi: 10.3390/jfmk10040377 (PMC12551099; doi:10.3390/jfmk10040377)
Supplement: Supplementary file 1 [file jfmk-10-00377-s001.zip › jfmk-3869763-supplementary.pdf]

Table S1: 95% Confidence interval (CI) for PM correlation (nonparametric bootstrap procedure) matrix: women vs. men. Each cell in the matrix includes the r value and the 95% CI in women (top) and men (bottom).

Women / Men	Fcon	95%confidence interval		Fecc	95%confidence interval		Econ	95%confidence interval		Eecc	95%confidence interval		Ucon	95%confidence interval		Uecc	95%confidence interval		Rcon	95%confidence interval		Recc
Fcon	1.00																					
	1.00																					
Fecc	0.929**	0.776	0.984	1.00																		
	0.978**	0.937	0.992	1.00																		
Econ	-0.27	-0.593	-0.023	-0.15	-0.480	0.143	1.00															
	0.554*	0.206	0.817	0.538*	0.227	0.799	1.00															
Eecc	-0.32	-0.698	-0.003	-0.229	-0.601	0.094	0.947**	0.863	0.977	1.00												
	0.693**	0.473	0.874	0.677**	0.441	0.876	0.886**	0.782	0.945	1.00												
Ucon	0.500*	-0.214	0.822	0.467*	-0.174	0.819	-0.14	-0.500	0.123	-0.05	-0.488	0.226	1.00									
	0.702**	0.426	0.866	0.666**	0.404	0.843	0.590**	0.183	0.839	0.625**	0.334	0.813	1.00									
Uecc	0.488*	-0.221	0.812	0.515*	-0.025	0.829	-0.11	-0.463	0.163	-0.03	-0.422	0.244	0.966**	0.872	0.994	1.00						
	0.714**	0.451	0.862	0.691**	0.430	0.853	0.551*	0.158	0.812	0.598**	0.291	0.793	0.990**	0.972	0.997	1.00						
Rcon	0.31	-0.126	0.631	0.25	-0.283	0.592	-0.07	-0.623	0.533	-0.097	-0.641	0.542	0.35	-0.027	0.594	0.38	0.015	0.650	1.00			
	0.27	-0.149	0.620	0.26	-0.177	0.619	0.30	-0.112	0.619	0.35	-0.022	0.632	0.41	0.111	0.731	0.36	0.043	0.671	1.00			
Recc	0.27	-0.152	0.589	0.21	-0.264	0.542	-0.05	-0.649	0.574	-0.08	-0.633	0.543	0.38	0.023	0.603	0.40	0.043	0.656	0.960**	0.920	0.984	1.00
	0.15	-0.219	0.563	0.11	-0.282	0.542	0.24	-0.178	0.592	0.32	-0.022	0.609	0.31	0.000	0.684	0.26	-0.067	0.643	0.926**	0.863	0.979	1.00

*p<0.05, **p<0.01, **p<0.001.

Index: con – concentric, ecc – eccentric, F - flexors, E - extensors, U - ulnar deviators, R - radial deviators, PM - peak moment.
